# Reflection on design and testing of pancreatic alpha-amylase inhibitors: an *in silico* comparison between rat and rabbit enzyme models

**DOI:** 10.1186/2008-2231-20-77

**Published:** 2012-11-20

**Authors:** Shiva Khalil-Moghaddam, Azadeh Ebrahim-Habibi, Parvin Pasalar, Parichehreh Yaghmaei, Nasim Hayati-Roodbari

**Affiliations:** 1 Biology Department, Science and Research Branch, Islamic Azad University, Tehran, Iran; 2 Endocrinology and Metabolism Research Center, Tehran University of Medical Sciences, Tehran, Iran; 3 Department of Biochemistry, Faculty of Medicine, Tehran University of Medical Sciences, Tehran, Iran

**Keywords:** Rat alpha-amylase, Rabbit alpha-amylase, Amylase inhibitor, Homology modeling, Docking

## Abstract

**Background:**

Inhibitors of pancreatic alpha-amylase are potential drugs to treat diabetes and obesity. In order to find compounds that would be effective amylase inhibitors, *in vitro* and *in vivo* models are usually used. The accuracy of models is limited, but these tools are nonetheless valuable. *In vitro* models could be used in large screenings involving thousands of chemicals that are tested to find potential lead compounds. *In vivo* models are still used as preliminary mean of testing compounds behavior in the whole organism. In the case of alpha-amylase inhibitors, both rats and rabbits could be chosen as *in vivo* models. The question was which animal could present more accuracy with regard to its pancreatic alpha-amylase.

**Results:**

As there is no crystal structure of these enzymes, a molecular modeling study was done in order to compare the rabbit and rat enzymes with the human one. The overall result is that rabbit enzyme could probably be a better choice in this regard, but in the case of large ligands, which could make putative interactions with the −4 subsite of pancreatic alpha-amylase, interpretation of results should be made cautiously.

**Conclusion:**

Molecular modeling tools could be used to choose the most suitable model enzyme that would help to identify new enzyme inhibitors. In the case of alpha-amylase, three-dimensional structures of animal enzymes show differences with the human one which should be taken into account when testing potential new drugs.

## Background

Carbohydrate digestion has been targeted as a mean to control both postprandial increase of blood glucose and weight gain [1]. Inhibitors of carbohydrate digesting enzymes, such as alpha-amylase and alpha-glucosidase, are now actively searched for, since they could ultimately make useful medicines against diabetes and obesity [2,3]. There are numerous examples where inhibitors that were found to be effective on glycosidic enzymes *in vitro*[4-7], proved to possess hypoglycemic and weight decreasing effect *in vivo*[5,7-9]. Pancreatic alpha-amylase inhibitors could be foreseen to become part of the drugs used for the so-called “diabesity” state. This enzyme inhibitors are especially of interest, as they have been reported to be devoid of the disturbing gastro-intestinal side-effect of some alpha-glucosidase inhibitors [10,11].

Although the final target of this compounds is usually the human pancreatic alpha-amylase, it is still common place to use similar enzymes as models in the *in vitro* studies [5,6]. In the next level, use of animal models is also a common method to assess the effect of these compounds, and in most of these studies, rats are privileged, as accessible, established and reproducible models of diabetes and obesity. There are also other alternatives, such as the possibility of using rabbits [12], it would be thus interesting to see which animal’s pancreatic enzyme is more similar to the human one, and how this similarity/ difference would affect the possibility of extrapolating the outcome of animal tests to human beings. Since there is no crystal structure of rat and rabbit enzymes, computer-generated models were used in this study in order to compare rabbit, rat, and human pancreatic enzymes with regard to their binding to a carbohydrate-based ligand as mimic of an inhibitor.

## Methods

### Sequences alignments

Sequences of rabbit (*Oryctolagus cuniculus*_protein accession number: XP_002715871) and rat (*Rattus norvegicus*_protein accession number: P00689) enzymes were retrieved from the NCBI (protein) (http://www.ncbi.nlm.nih.gov). The 3OLD.pdb file of the human enzyme was used to assess the length of the signal peptide, which was omitted in the alignments. ClustalW [13] and the BLAST tool of the NCBI site were used to perform alignments. The BLAST program compares the protein sequences and calculates the statistical significance of sequences that are matched together, and reports the percentage of “identity” (residues that are identical) and “similarity” (residues that are conserved), which have been mentioned in the Results section. Clustal W was used to make a multiple alignment in order to have an insight into areas of similarity (usually associated with conserved functional domains) as well as differences that occur as a consequence to substitutions and deletions of amino acids.

### Model generation

The ModWeb server version SVN.r1368M (web based version of Modeller [14]) was used in order to make the rabbit and rat pancreatic amylase model (https://modbase.compbio.ucsf.edu/scgi/modweb.cgi). Modeller performs comparative modeling by satisfaction of spatial restraints [15]. The program uses the protein sequence, and a three-dimensional structure that has a high enough similarity to the sequence to make a three-dimensional protein model. In this case 1hx0.pdb corresponding to a porcine alpha-amylase with 1.38 Å resolution was used. Usually, 30% of identity between template and target is sufficient to obtain a reliable model [16]; in this case, percentage of identical residues of human and porcine enzymes with rat and rabbit is higher than 80%. Although multiple alpha-amylase structures have been elucidated, at the time this study has been done, the 1hx0.pdb structure had the best resolution between available mammalian alpha-amylase structures. The sequence of rat and rabbit alpha-amylase were given to the server, and the resulting models were retrieved. Model quality was assessed with the Molprobity [17] server, which analyses side chain rotamers and provide Ramachandran plots, which are indicative of the geometrical parameters of the model.

### Docking and molecular dynamics simulation

Docking was performed with Autodock vina [18]. The 3OLI.pdb file was first processed with the use of MOE 2009.10 (Chemical Computing Group Inc., Montreal,Canada). Additional molecules to alpha-amylase, including solvent, were deleted prior to docking. MGLtools (v. 15.4, revision 24) which is the graphical interface to Autodock was used to define the docking box and assign gasteiger charges to protein and ligand molecules. The docking box was positioned at x = −8.548, y = −21.483, z = 18.925 with a size of 64x82x58. A configuration file was prepared as the input of Autodock vina. In addition to the docking box specifications, in this configuration file, exhaustiveness (related to the docking precision and usually set at 8) was set on 20 and 100 poses were generated for the ligand. To validate the docking method that was used, RMSD between actual pose of the co-crystallized ligand and docked one was assessed with the use of the Profit server (http://www.bioinf.org.uk/software/profit) which is based on the McLachlan algorithm [19]. The default method was used with inclusion of heteroatoms only and calculating RMSD for all atoms.

The dynamics simulation module of MOE 2009.10 was used to perform a short simulation of 5000 picoseconds length with T = 300K using the Noise-Poicare-Andersen Hamiltonian equations of motion. The protein was first minimized. Then solvation was done with 17725 water molecules. A preliminary simulation of t = 100 picoseconds, T = 300K was performed to equilibrate the system, and the output was used as the input of the actual 5000 picoseconds run. Sampling was done at each 5 picoseconds. The MMFFx94 forcefield was used for all the calculations.

## Results and discussion

**Figure 1:**
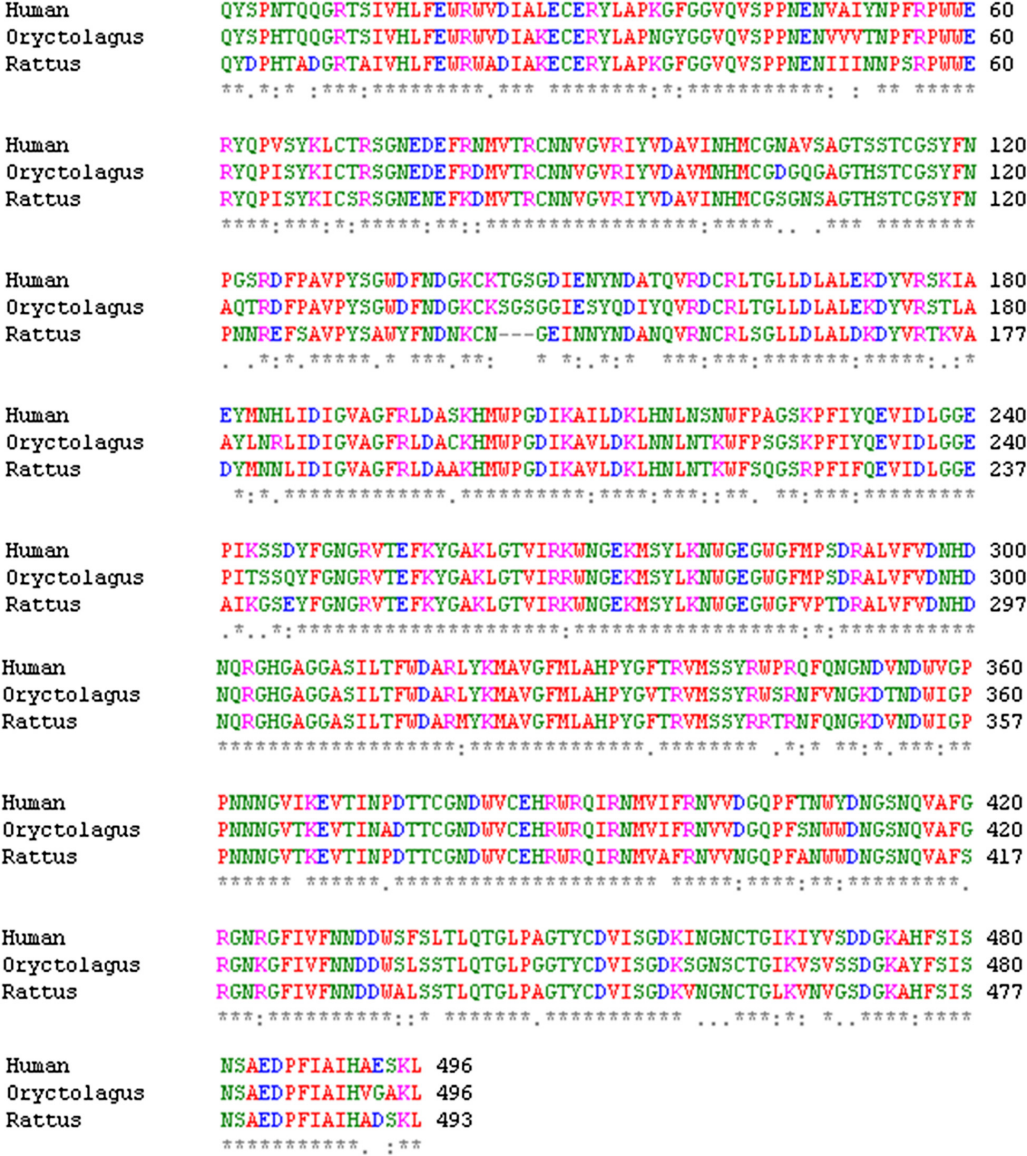
**Results of the ClustalW [**[13]**] alignment for the pancreatic alpha-amylase amino acid sequences of human, rabbit (*Oryctolagus cuniculus*), and rat (*Rattus norvegicus*) enzymes.** Conserved regions are shown with asterisk. Colour codes of amino acids are as follows: residues A,V,F,P,M,I,L, and W (small + hydrophobic (incl.aromatic -Y))in red, residues Dand E (acidic) in blue, residues R and K (basic) in magenta, residues S,T,Y,H,C,N,G, and Q (Hydroxyl + sulfhydryl + amine + G) in green.

The first stage in the process of choosing the more suitable model between rat (RPA) and rabbit (RABPA) enzymes is the comparison of their amino acid sequences with the human one (HPA). Figure 1 shows the result of a multiple alignment between the three sequences. A pairwise alignment performed with BLAST reveals that the rat and human enzyme have 84% identities and 92% similarity. For the rabbit enzyme these figures become 87% and 92% respectively. However, beside the higher identities of the rabbit and human enzyme, it is interesting to notice the presence of a gap in the rat sequence, which is located at the position of amino acids 143–145 of HPA and RABPA. This gap makes the rat alpha-amylase 3 residues shorter than the human and rabbit one. The same gap is seen in the mouse sequence. This sequence is TGS in HPA and SGS in RABPA, which could be estimated to be conserved between human and rabbit enzymes. The TGS sequence is also detected in primates (e.g. *Macaca mulatta*, *Gorilla gorilla*), *Equus caballus*, *Bos taurus*, *Canis familiaris*, *Taeniopygia guttata, monodelphis domestica, Danio rerio*, *Myxocyprinus asiaticus*, and *Xenopus tropicalis*; as TAS in *Sus scrofa*, *Meleagris gallopavo*, and *Gallus gallus*, and as TYS in *Anolis carolinensis*.

In the next stage, a three-dimensional model was made for RPA and RABPA. It should be mentioned that the tertiary structure of mammalian alpha-amylase has been reported only for human salivary and pancreatic enzyme, as well as porcine pancreatic enzyme. Small differences in the spatial arrangement of residues (in similar enzymes) could produce large effects on their interactions with potential ligands [20], and the fact that proteins belong to the same family is not enough to predict their ligand binding profile [21]. In the absence of an experimentally obtained structure, the best way to detect these differences is to study a model. Protein models that are made by comparative modeling are considered to be highly reliable, especially when the percentage of identical residues is elevated [16]. The template used to model RPA and RABPA was the 1hx0.pdb [22] structure which originally contained a carbohydrate-based ligand derivated from acarbose. As mentioned in the Methods section, this structure had the highest resolution in mammalian alpha-amylases structures of the protein database (PDB), at the time this study was performed. No missing atoms were detected in this template. Ramachandran plot of 1 h × 0.pdb indicates that 100% of its residues are situated in the allowed region. RPA and RABPA models were generated by the ModWeb server and subsequently minimized in MOE. Quality assessment of the generated models indicated them to be reliable. First, the results of evaluation methods that are implemented within the ModWeb server output were acceptable. The GA341 score was 1.0 for both models which is indicative of a reliable fold (GA341 > 0.7 is related to ≥ 95% probability of correct fold). The MPQS (ModPipe Quality Score) was 1.96 for RABPA and 2.08 for RPA which is a further approval of the reliability of the model (MPQS value higher than 1.1 is indicative of a reliable model) [14]. Furthermore, as a mean to assess the geometrical validity of the models. Ramachandran plots [23] of RABPA and RPA were obtained from the MolProbity server. Ramachandran graphs of the two models indicated that for RABPA 99.8% (492/493) of all residues were in allowed regions and the only outlier was N378, where RPA had 100% (490/490) of all its residues in the allowed region, with no outlier in the structure. HPA, RPA and RABPA possess five conserved disulfide bonds, which correspond to residues pairs 28–86, 70-115, 141–160, 378–384, 450–462 in HPA and RABPA, and residues pairs 28–86, 70-115,141-157,375-381, and 447–459 in RPA, in which the numbering is altered due to the three-residue gap. It should be mentioned that the numbering here has been made according to the putative expressed protein (with omission of the signal peptide, by comparison with HPA_see Figure 1). In light of these quality assessment results, and with regard to the high similarity of the templates with the modeled targets, we believe that these models could be considered to have enough accuracy and biological plausibility for further ligand binding studies.

**Figure 2:**
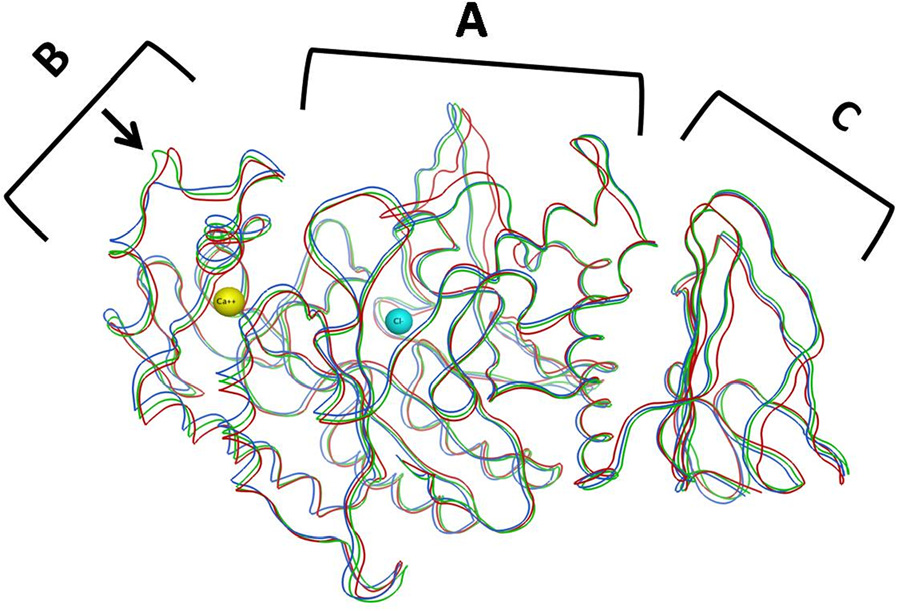
**Superimposed backbone structures of human (red ribbons), rabbit (green ribbons), and rat (blue ribbons) pancreatic alpha-amylases.** Human structure is obtained from the 3OLI.pdb file, and rabbit and rat enzymes are modeled. The three main domains of the enzymes are indicated (A,B, and C). The two spheres are one calcium (in the left side of the picture, yellow) and one chloride atom (in the center, cyan), which are located in their conserved position among all three enzymes. The arrow indicated the shorter loop of the rat enzymes which lacks residues 143–145 of human and rabbit enzymes.

**Figure 3:**
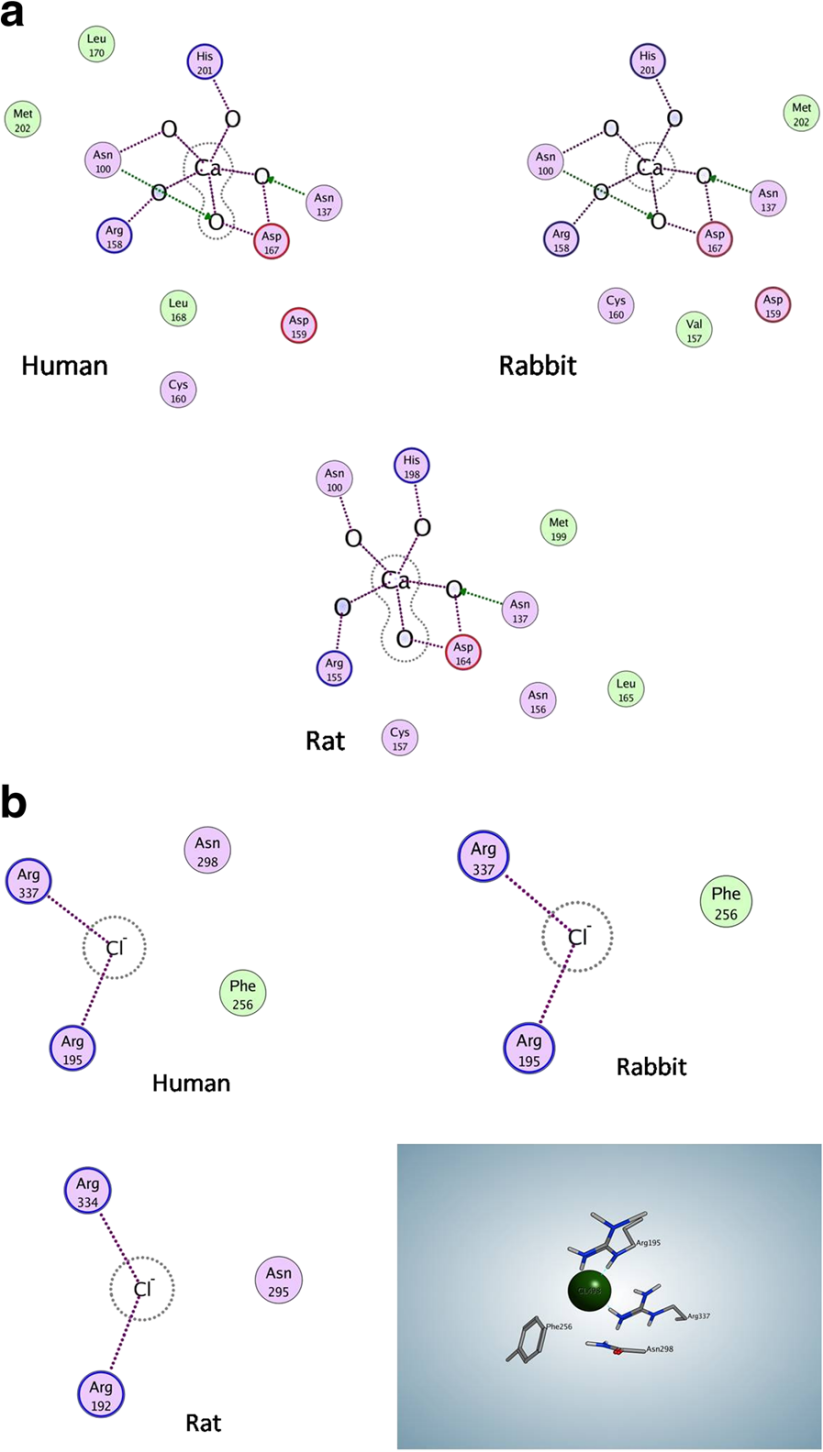
**a. Coordination state of the calcium ion as found in the three-dimensional structures of human enzyme, as well as in rabbit and rat enzymes.b**. Interactions observed between the chloride ion of human, rabbit, and rat enzymes. The ligand interaction module of MOE.2009.10 has been used to obtain the two-dimensional schematic representation of the residues positioned in the vicinity of the ions. In order to provide a better feeling of the three-dimensional arrangement of the shown residues in the case of chloride, this site is shown in rabbit enzyme, with chloride ion shown as a sphere, and nearby amino acids as ball and sticks. Colour codes of amino acids are as follows: basic residues in pink with blue border, acidic residues in pink with red border, polar residues in pink, hydrophobic (greasy) residues in green.

The superimposed minimized backbone structures of the three enzymes are shown in Figure 2, where the three domains of the enzymes (A-C) are indicated. Positioning of the calcium and chloride ions are related to the human one, and could be assumed to be similar in RPA and RABPA. The nearby residues interacting with these ions are shown in Figure 3a and b for comparison means and indicate no major difference between the three enzymes, a fact that is a further indication of the biological plausibility of the models. The calcium ion of HPA makes its interactions with the residues of domain B, with the exception of His201 which belongs to Domain A [20]. This ion is considered to be “a central organizing structural feature of Domain B” [24], is needed for alpha-amylase catalytic activity, and its binding pocket is essential to maintain the fold stability [25]. The chloride ion was previously thought to be a feature of mammalian amylase, and shown to possess activating property [26]. In a thorough study to characterize the chloride binding pocket of alpha-amylases, the anion was found to be coordinated by one arginine, one arginine/lysine and an asparagine residue throughout chloride-dependent amylases, with R337 (PPA numbering) being a key feature for the anion binding [27].

The three missing residues detected in the rat sequence are found to be located in a loop of domain B, which makes the loop distinctively shorter in RPA (Figure 2).

**Figure 4:**
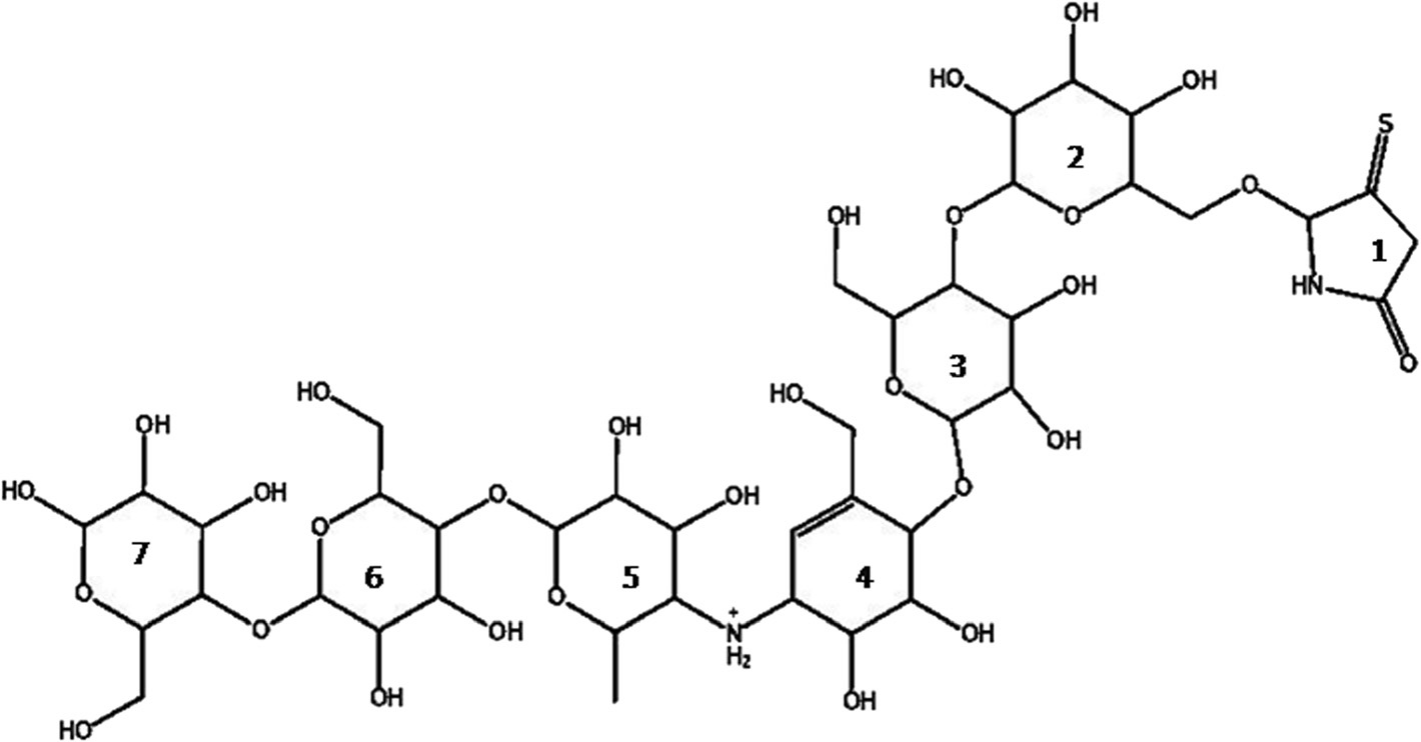
**Scheme of the seven-ring containing ligand containing an acarviostatin derivative and a thiohydantoin-like moiety.** Numbering of the rings is arbitrary and has been done in order to facilitate description of its interactions.

In the next stage, a putative inhibitor was designed to assess the differences of active site ligand binding between the two models. As previously mentioned, acarviostatin-derived products have been reported as potent inhibitors of HPA, and occupancy of seven subsites of HPA active site suggested to be an efficient mean to inhibit the enzyme [28]. On the other hand, acarviosinyl-isomaltosyl-spiro-thiohydantoin has also been previously designed and tested as a potent inhibitor of mammalian amylase [29]. Thus, a hybrid compound structure was designed which contained these two potent moieties. The ligand is composed of a six carbohydrate-based units (as found in the crystal structure 3OLI.pdb), with an added moiety resembling thiohydantoin (Figure 4) in order to be able to fill the seven subsites. Thiohydantoin contains one additional nitrogen atom that was omitted here in order to make a more hydrophobic structure. The compound was manually implemented in the 3OLI.pdb structure by superimposition to the existing ligand. However, a further testing was done with a docking tool (Autodock vina) in order to have an approval for the location of the ligand. The docking method was first assessed with a docking experiment on the crystallized ligand. An RMSD of 1.9 Å was obtained between the best pose obtained by docking and the actual binding mode. This is satisfactory with regard to the less than 2 Å threshold usually used to assess successful docking [30], and especially with regard to the large size and multiple rotatable bonds of the ligand. Results of the proposed compound docking showed that the first docked pose was comparable to the manually positioned ligand, but the latter was further used, since derived from a crystallographically obtained pose.

In order to get an approximation of the possible effectiveness of this ligand as a potential inhibitor of the enzyme, docking score was obtained for the HPA co-crystallized pseudo saccharide ligand acarviostatin II03 (with 7 carbohydrate-based units). This score was −14.6 kcal/mol for acarviostatin II03 while our ligand achieved a −13.4 kcal/mol. In comparison, acarbose (with four units) had a score of −11 kcal/mol. The K_i_ of acarviostatin II03 is reported to be of 0.0147 μM and the K_i_ of acarbose that is around 2.6 μM [28], which is suggesting that our ligand could be potentially better than acarbose, but this assumption remains to be verified.

**Figure 5:**
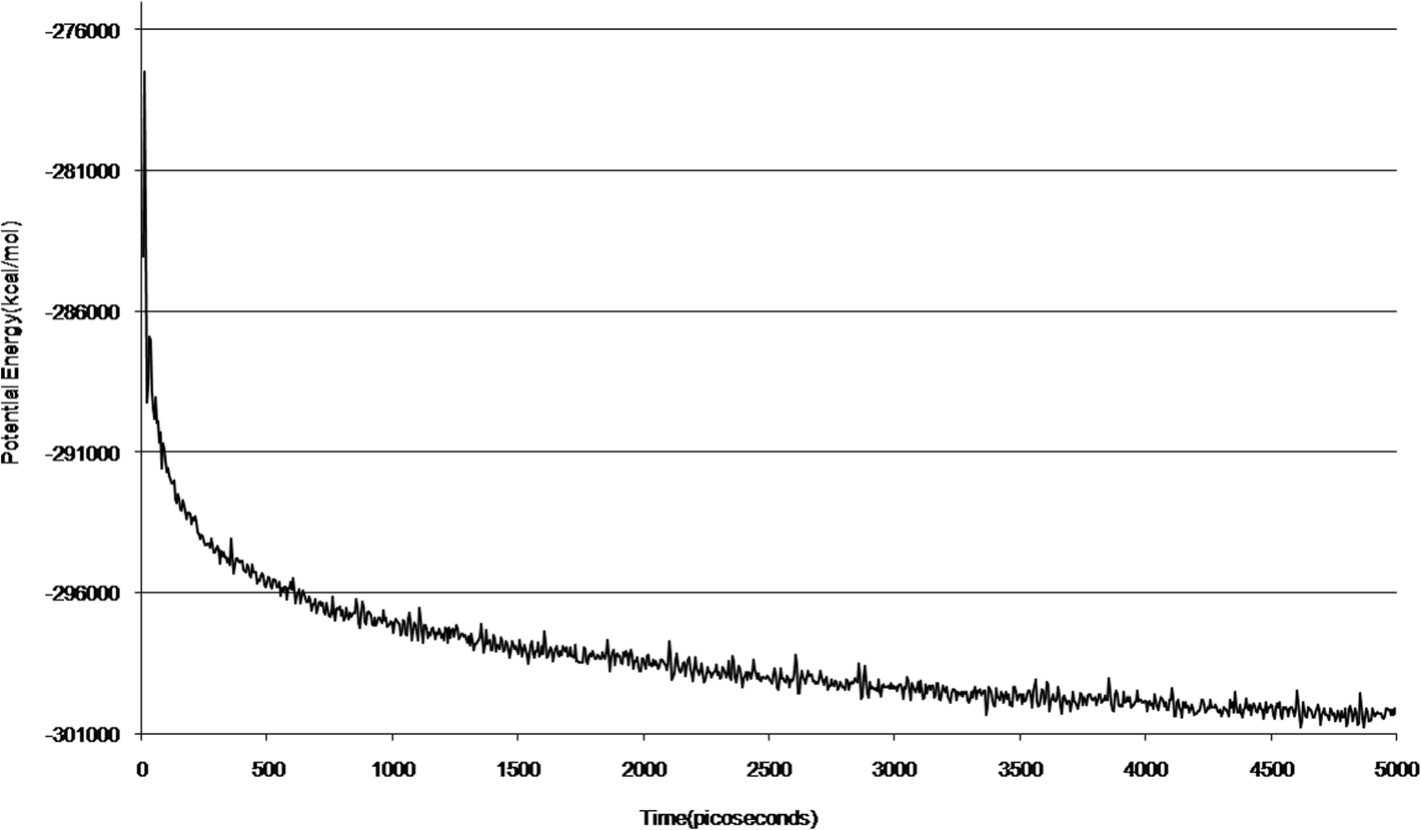
Results of the potential energy versus time, obtained for a 5000 picoseconds molecular dynamics simulation of human pancreatic amylase containing the seven-ringed ligand.

A short molecular dynamics simulation was then performed to get an insight of the interaction of the ligand with flexible active site residues in an aqueous environment. It should be mentioned that this simulation was ran as an alternative to flexible docking, for 5000 picoseconds which is acceptable in these cases [31,32]. The system potential energy is shown in Figure 5 and is indicative of an equilibrated stage in the last 1000 picoseconds.

**Figure 6:**
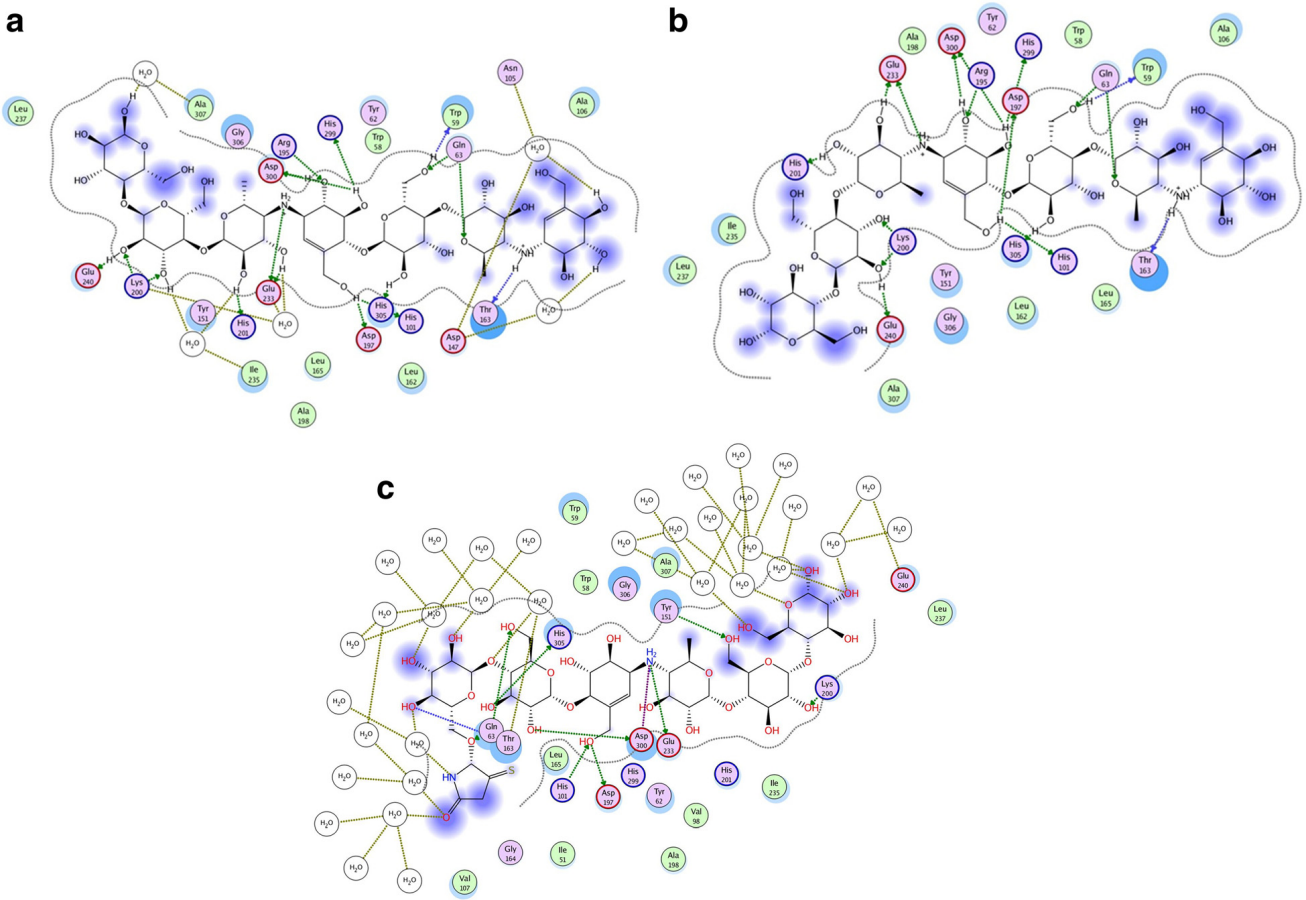
**Acarviostatin AIV03-derivated ligand interactions within the human enzyme in the 3OLI.pdb files.** Interaction diagrams were drawn with the use of MOE.2009.10 ligand-interaction module. Water molecules of the 3OLI.pdb structures have been preserved (**6.a**) or deleted (**6.b**) in order to assess their potential significance in ligand-protein interactions. Water molecules that are in the vicinity of amino acids are as HOH 586 (E233, catalytic residue), HOH 791 (N105),HOH 817 (I235) and HOH 832 (A307). Interaction between residues and ligand moieties are hydrogen bonds (indicated by arrows, green arrows indicate side chain donors and acceptors and blue arrows indicate backbone donors and acceptors). Colour codes of amino acids are as follows: basic residues in pink with blue border, acidic residues in pink with red border, polar residues in pink, hydrophobic (greasy) residues in green.

The seven active cleft subsites occupied by the original ligands span from the −4 subsite to the +3 one. Based on *in vitro* inhibition studies on acarviostatin derivatives, the −4 subsite has been particularly highlighted as a location whose occupation would increase the potency of designed inhibitors [18]. It is interesting to note that interaction of the original ligand with both −4 and +3 subsites occurs mainly through water molecules. Figure 6 shows an interaction diagram obtained with MOE.2009.10 when the solvent molecules of the 3OLI.pdb file were present or absent. As observed in Figure 6a, interaction of the ligand with subsite −4 N105 and D147 occurs via water molecules. Similarly, subsite +3 interactions are also happening via water molecule (in this structure). When water molecules are deleted, and the interaction diagram redrawn (Figure 6b), the two ending moieties of the ligand show no particular interactions.

**Figure 7:**
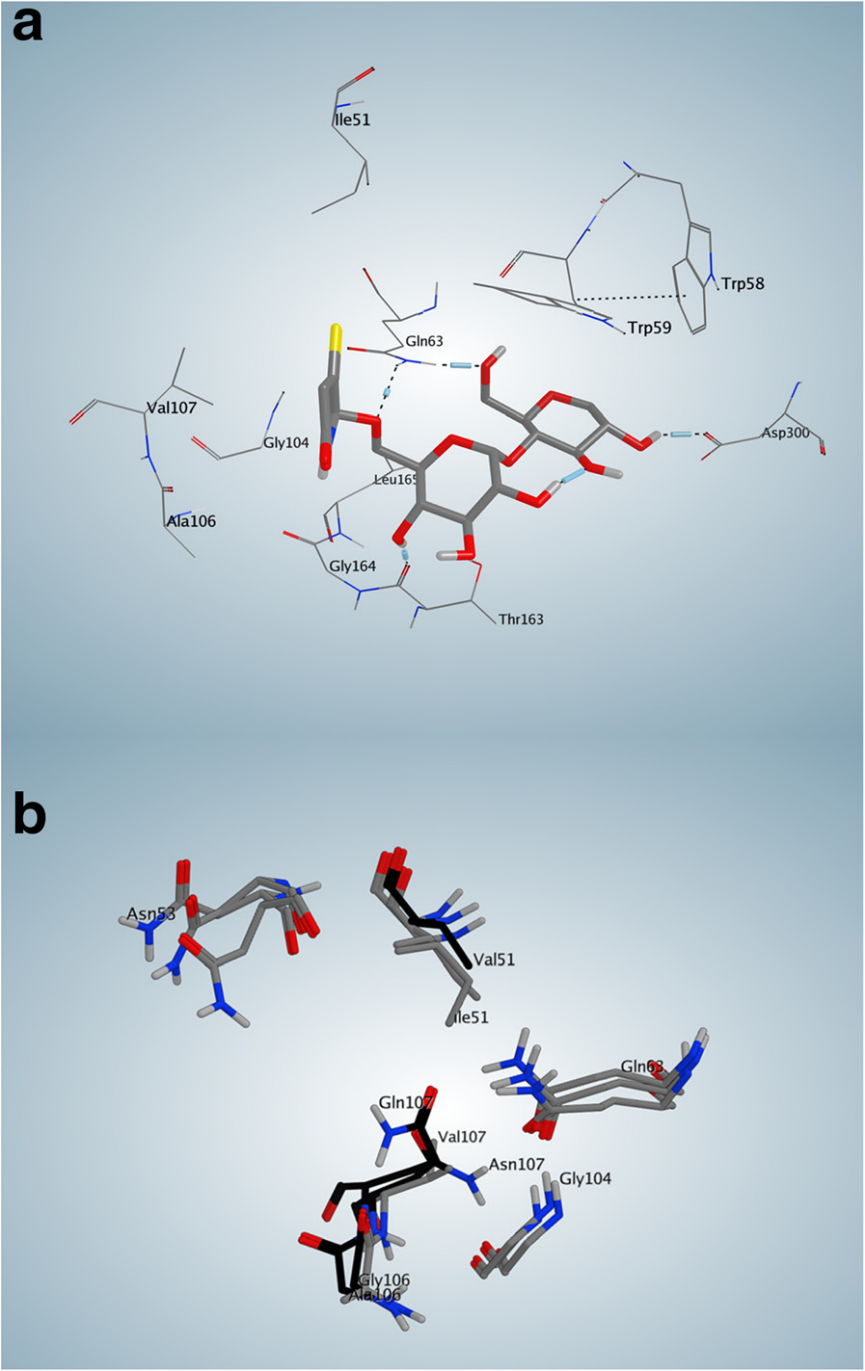
**Interactions of the seven-ringed ligand with subsite −4 of the three pancreatic enzymes.** Colour codes of amino acids are as follows: basic residues in pink with blue border, acidic residues in pink with red border, polar residues in pink, hydrophobic (greasy) residues in green. **a**. Interactions obtained with the ligand after 5000 picoseconds of simulation. The water network seems to be of importance in the interactions between ring number 1 and subsite −4 of the human enzyme. **b**. Residues found in the vicinity (5Å) of the seven-ringed ligand in human pancreatic enzyme. c. Superimposition of the interacting residues found in human enzyme, with the ones of rabbit and rat enzymes. Residues shown in black are different with the human enzyme in the rabbit and rat models.

In the case of our ligand, the interactions that occur in the final frame of the simulation are shown in Figure 7a. Residues related to subsite −4 that surround ring 1 of the ligand (see Figure 4) include I51, Q63, G104, A106, V107 and G164 (shown in Figure 7b) and seem to make a hydrophobic pocket. The hydrogen bond that occurs between Q63 and the ligand, as well as the interaction with T163 are also of interest. In order to compare the corresponding residues of RABPA and RPA enzymes related to the subsite −4, a superimposition of the most important residues was done (Figure 7c).

Residues N53, Q63 and G104 are conserved in all three enzymes. However, V51 replaces I51 in RABPA, but it is conserved in RPA as I51. As observed in Figure 7b, this makes a shorter side chain in this place for RABPA, and consequently, more space exists in this location. Instead of A106, both RABPA and RPA possess G106, again with the consequence of providing more available space in that position. The most important difference occurs in V107, where it is changed to Q107 in RABBPA and N107 in RPA. This is a more radical change, which results in polar, and bulkier side chains taking the place of an aliphatic side chain. Thus, a potential designed ligand possessing a bulkier moiety in place of ring 1 of our ligand could probably fail to interact properly in this region with RABPA or RPA, while it might have in fact a good positioning in the human enzyme. Reversely, if some designed ligand find enough space in the vicinity of residues 106 or 51 in RABPA or RPA, it may be too large for HPA. A review of the other residues that are interacting with rings 2–7 of the ligands (based on the diagram shown in Figure 7a) shows their perfect conservation in the three enzymes, with the exception of T163 being replaced by a serine residue in RPA, while remaining T163 in RABPA.

## Conclusion

Overall, the structural differences between RPA and HPA could be assessed to be more important than the ones between RABPA and the human enzyme, with regard to inhibitor design. Especially, the shortening of a loop in domain B of rat enzyme should be highlighted, as it may produce long-range effect in case of conformational changes. About the active site itself, if ligands are to be designed that would span subsites −3 to +3, RABPA would be a better choice for testing the compounds, and consequently, rabbit would be a better model in this regard. If a ligand present a structure capable of interacting with subsite −4, then results obtained from the use of both RABPA and RPA (and the animals themselves) could be different with the results obtained for the human enzyme. Since modeling techniques have now evolved to become more accessible, it would be advisable to use them in order to assess the possible positive/negative outcome of tests in animal models.

## Competing interests

The author state to have no conflicts of interests.

## Authors’ contributions

This work is the result of part of SK-M Ph.D. thesis, AE-H. and PY were the supervisors of this thesis and have provided the idea and guided the student throughout her work as well as in preparation and revision of the manuscript, and PS was an advisor as to biochemical concepts, NH-R has provided advisory help relative to physiological concepts. All authors read and approved the final manuscript.
